# Water- and Fat-Soluble Antioxidants in Human Seminal Plasma and Serum of Fertile Males

**DOI:** 10.3390/antiox8040096

**Published:** 2019-04-11

**Authors:** Giacomo Lazzarino, Ilaria Listorti, Gabriele Bilotta, Talia Capozzolo, Angela Maria Amorini, Salvatore Longo, Giuseppe Caruso, Giuseppe Lazzarino, Barbara Tavazzi, Pasquale Bilotta

**Affiliations:** 1 Institute of Biochemistry and Clinical Biochemistry, Catholic University of Rome, Largo F. Vito 1, 00168 Rome, Italy; giacomo.lazzarino@unicatt.it; 2 Fondazione Policlinico Universitario A. Gemelli IRCCS, Largo A. Gemelli 8, 00168 Rome, Italy; 3 Alma Res Fertility Center, Centro di Fecondazione Assistita Alma Res, Via Parenzo 12, 00199 Rome, Italy; laboratorio@almares.it (I.L.); bilotta.oblomov@gmail.com (G.B.); taliacapozzolo@almares.it (T.C.); pasquale.bilotta@almares.it (P.B.); 4 Department of Biomedical and Biotechnological Sciences, Division of Medical Biochemistry, University of Catania, Viale A. Doria 6, 95125 Catania, Italy; amorini@unict.it; 5 LTA-Biotech srl, Viale Don Orione, 3D, 95047 Paternò (CT), Italy; salvo.longo@hotmail.it; 6 Oasi Research Institute—IRCCS, Via Conte Ruggero, 73, 94018 Troina (EN), Italy; forgiuseppecaruso@gmail.com

**Keywords:** water-soluble antioxidants, fat-soluble antioxidants, human seminal plasma, human serum, reactive oxygen species, oxidative/nitrosative stress, male infertility

## Abstract

Reactive oxygen species (ROS) are physiologically involved in functions like sperm maturation, capacitation and acrosome reaction, but their excess is involved in male infertility. Antioxidants in seminal plasma (SP) are an important factor balancing physiologic and harmful ROS activities. In this study, we determined and compared the full profiles of the water- and fat-soluble antioxidants in SP and serum of 15 healthy fertile subjects (ranging between the ages of 35 and 42 years). Ejaculates were obtained after 2–5 days of sexual abstinence. After liquefaction and withdrawal of an aliquot for the sperm count, samples were centrifuged to obtain SP. Thirty min after semen donation, a venous blood sample was collected from each subject. Donors with lower SP concentrations of ascorbic acid (*n* = 5) or α-tocopherol (*n* = 5) received a 4 week oral administration of either vitamin C (100 mg/day) or vitamin E (30 mg/day). They were then re-assayed to determine the SP and serum levels of ascorbic acid and α-tocopherol. SP and serum samples were properly processed and analyzed by HPLC methods suitable to determine water (ascorbic acid, glutathione (GSH) and uric acid) and fat-soluble (all-*trans*-retinoic acid, all-*trans*-retinol, α-tocopherol, carotenoids and coenzyme Q_10_) antioxidants. Data demonstrate that only ascorbic acid is higher in SP than in serum (SP/serum ratio = 4.97 ± 0.88). The other water-soluble antioxidants are equally distributed in the two fluids (GSH SP/serum ratio = 1.14 ± 0.34; uric acid SP/serum ratio = 0.82 ± 0.12). All fat-soluble antioxidants are about 10 times less concentrated in SP than in serum. In donors treated with vitamin C or vitamin E, ascorbic acid and α-tocopherol significantly increased in both fluids. However, the SP/serum ratio of ascorbic acid was 4.15 ± 0.45 before and 3.27 ± 0.39 after treatment, whilst those of α-tocopherol were 0.11 ± 0.03 before and 0.10 ± 0.02 after treatment. The results of this study, by showing the peculiar composition in water- and fat-soluble antioxidants SP, indicate that it is likely that still-unknown mechanisms allow ascorbic acid accumulation in SP against a concentration gradient. SP mainly relies its defenses on water- rather than fat-soluble antioxidants and on the mechanisms ensuring their transfer from serum.

## 1. Introduction

Oxidative stress is considered an imbalance between reactive oxygen species (ROS) production and cell antioxidants [1]. In the normal healthy state, the generation of ROS occurs in a controlled manner, with mitochondria as the main intracellular source of ROS in the form of superoxide anions through the electron transport chain [2]. Increased ROS production occurs under high-stress conditions or in various disease states such as acute [3,4] and chronic neurodegenerations [5], stroke [6], ischemia [7] and male infertility [8]. These pathologic conditions are characterized by malfunctioning mitochondria, which are unable to correctly catalyze the tetravalent reduction of molecular oxygen to water in the electron transport chain, leading to increased ROS generation from aerobic respiration. Furthermore, most of the aforementioned pathologies trigger inflammatory processes [9] which are additional relevant sources of ROS [10]. Oxidative stress causes cumulative oxidative damages to macromolecules [11,12,13,14] and is responsible for cell death via both apoptosis [15] and necrosis [16]. In various biological contexts, it has been demonstrated that oxidative stress is associated with nitrosative stress [17], caused by an excess production of reactive nitrogen species (RNS). The coincidence of these phenomena, defined as oxidative/nitrosative stress, is capable of generating peroxynitrite (ONOO^−^•), one of the most dangerous free radical molecules for the biological systems.

It is well known that any biological system is equipped with both enzymatic and non-enzymatic systems scavenging for these harmful compounds. Non-enzymatic antioxidants are low molecular weight compounds that, thanks to their high reducing power, are capable of scavenging indiscriminately for any type of ROS. They have different chemical structures and can be classified into two gross divisions, depending on their solubility in water (hydrophilic) or fat (hydrophobic). Generally, water soluble antioxidants react with ROS within cells or body fluids (blood serum, extracellular fluid, seminal plasma) while fat-soluble antioxidants are more prone to protect cell membranes from ROS-mediated lipid peroxidation. Humans synthesize only a few low molecular weight antioxidants, the main antioxidants being reduced glutathione (GSH), uric acid and coenzyme Q_10_. Of these, only GSH has the primary role of functioning as a cell antioxidant [18]. In fact, coenzyme Q_10_ is mainly sequestered within mitochondria, acting as an electron transporter in the electron transport chain, and the activity of uric acid as an intracellular antioxidant is negligible since it is ~2000 fold less concentrated than GSH and other water soluble antioxidants [19]. The majority of low molecular weight antioxidants in the human body (ascorbic acid, tocopherols, carotenoids, polyphenols, etc.) are introduced through diet and distributed to organs and tissues through the bloodstream. In a given subject, the quantitative and qualitative determination of the circulating levels of low molecular weight antioxidants allows extrapolating whether defenses against potential ROS damages are high or low, and evidencing a possible deficit of specific antioxidant(s) [20].

Infertility, defined as the inability to achieve pregnancy after at least twelve months of regular intercourse, is a multi-factorial pathological state, affecting approximately 48.5 million couples worldwide [21]. Epidemiological studies demonstrated that a male factor is responsible in about 50% of cases of pregnancy failure [22,23,24]. Although male infertility is considered a multifactorial disease, with pathophysiological, environmental, genetic and lifestyle factors involved in its occurrence [25], ROS-mediated oxidative stress has been associated with the pathobiological processes of male infertility [26,27,28].

Notwithstanding, physiological ROS levels play important roles in proper sperm processes, such as maturation [29,30], capacitation [31], hyperactivation [32,33], acrosome reaction [34] and sperm-oocyte fusion [35]. It is, however, well demonstrated that oxidative stress causes spermatozoa lipid peroxidation [36], oxidative modifications of proteins [37] and oxidative damages to DNA [38]. Significant contributions against oxidative stress are certainly offered by both water- and fat-soluble low molecular weight antioxidants in seminal plasma [39]. The levels of most of these compounds in seminal plasma (SP) are strictly dependent on their relative concentrations in the blood stream, with these being dictated by the diet regimen followed by an individual [40].

This study was designed to determine the concentrations of hydrophilic and hydrophobic antioxidants in both human blood serum and SP of fertile subjects in order to better evaluate the specific antioxidant profiles of each fluid. Concentrations of biomarkers related to oxidative/nitrosative stress, such as 8-hydroxy-2′-deoxyguanosine (8-OHdG), malondialdehyde (MDA), nitrites (–NO_2_^−^) and nitrates (–NO_3_^−^) were also measured. Furthermore, the influence of administration of vitamins C and E on the SP concentrations of ascorbic acid and α-tocopherol was also evaluated.

## 2. Materials and Methods

### 2.1. Sampling of Serum and Seminal Plasma

This study was conducted according to the Declaration of Helsinki for Medical Research Involving Human Subjects (Ethical Committee approval: prot. 1F295.52-20/10/2017). Informed written consent was obtained from each subject enrolled in the study.

Ejaculates and peripheral venous blood samples were obtained from 15 healthy volunteers (ranging between the ages of 32 and 45 years), recruited among the personnel of the Catholic University of Rome. The intake of dietary supplements, beverages and foods rich in water- or fat-soluble antioxidants, alcohol or drug abuse and smoking were used as exclusion criteria. Conversely, proven fertility (presence of offspring) was used as the only inclusion criterion. In the interview, all participants affirmed to follow a balanced Mediterranean diet and to carry out mild-to-moderate physical activity.

Semen specimens were produced by masturbation after a recommended period of 2–5 days of sexual abstinence. After a complete liquefaction at 37 °C for 20 min, a spermiogram to determine sperm motility, concentration and morphology, according to World Health Organization (WHO) guidelines [41], was carried out. Subsequently, the light-protected liquefied semen samples were centrifuged at 1480× *g* for 10 min at room temperature and the upper SP was immediately withdrawn and processed for water- and fat-soluble antioxidant extractions [42,43]. Within 30 min of ejaculation, whole blood was collected from the antecubital vein into a VACUETTE polypropylene tube containing serum separator and clot activator (Greiner-Bio One GmbH, Kremsmunster, Austria) and immediately protected from light. After 30 min at room temperature in the dark, samples were centrifuged at 1890× *g* for 10 min at 10 °C and the separated sera were immediately withdrawn and processed for the extraction of water- and fat-soluble antioxidants [42,43].

A subgroup of five of the fertile healthy control group, who had the lowest SP and serum values of ascorbic acid, received an oral daily supplementation of 100 mg of vitamin C for 4 weeks, at the end of which they donated new semen and blood samples that were processed and analyzed to determine the concentrations of ascorbic acid in SP and serum. Similarly, a second subgroup composed by those who had the lowest SP and serum values of α-tocopherol received an oral daily supplementation of 20 International Units (IU) of α-tocopherol (30 mg) for 4 weeks, at the end of which they donated new semen and blood samples that were processed and analyzed to determine the concentrations of α-tocopherol in SP and serum.

### 2.2. Sample Processing and HPLC Analyses

Each SP and serum sample was divided into two aliquots in order to perform separate extractions of water- and fat-soluble antioxidants. The first aliquot of SP and serum (500 μL) was used for the extraction of hydrophilic antioxidants and biomarkers of oxidative/nitrosative stress, as described elsewhere [42]. Samples were briefly deproteinized by adding 1 mL of HPLC-grade CH_3_CN (VWR Chemicals, Briare, France) immediately centrifuged to pellet precipitated proteins and supernatants washed with HPLC-grade chloroform to remove organic solvent. The upper aqueous phases of serum and seminal plasma were diluted 3 and 10 times, respectively, with HPLC-grade water and then directly injected (100 μL) onto the HPLC Hypersil C-18, 250 × 4.6 mm, 5 μm particle size column (Thermo Fisher Scientific, Rodano, Milan, Italy), provided with its own guard column, for the analysis of ascorbic acid, GSH, uric acid, MDA, –NO_2_^−^, –NO_3_^−^ and 8-OHdG.

The processing to extract fat-soluble antioxidants was carried out on the second aliquot of SP and serum using a method recently described in detail elsewhere [43]. SP or serum (500 µL) was added to 1 mL of HPLC-grade CH_3_CN. After vigorous vortexing for 60 s, these mixtures were incubated at 37 °C for 1 h in a water bath under agitation to allow the full extraction of lipid soluble compounds, and then centrifuged at 20,690× *g* for 15 min at 4 °C to precipitate proteins. Clear supernatants were directly used for the reversed phase HPLC analysis of all-*trans*-retinoic acid, all-*trans*-retinol, astaxanthin, lutein, zeaxanthin, *trans*-β-apo-8′-carotenal, γ-tocopherol, β-cryptoxanthin, α-tocopherol, lycopene, α-carotene, β-carotene and coenzyme Q_10_ using a Hypersil Gold RP C18, 150 × 4.6 mm, 5 µm particle size column, provided with its own guard column (Thermo Fisher Scientific, Rodano, Milan, Italy). All the aforementioned procedures were carried out by protecting samples from light in order to avoid the degradation of photo-sensitive molecules.

For both analyses, the HPLC was a Spectra System P4000 pump, equipped with a highly sensitive 5 cm light-path flow cell UV6000LP diode array detector, set up for acquisition between 200 and 550 nm wavelengths (Thermo Fisher Scientific, Rodano, Milan, Italy). Data acquisition and analysis were carried out using the ChromQuest software provided by the HPLC manufacturer.

Identification and quantification of the different compounds in chromatographic runs of SP and serum samples were performed by comparing retention times and absorption spectra of the various peaks with those of runs of ultrapure standard mixtures with known concentrations. In the final calculations, the sum of the concentrations of astaxanthin, lutein, zeaxanthin, *trans*-β-apo-8′-carotenal, β-cryptoxanthin, lycopene, α-carotene and β-carotene was performed, and the fat-soluble antioxidants were reported hereinafter as total carotenoids.

### 2.3. Statistics

Comparison of the concentrations of the compounds of interest recorded in the two biological fluids was performed by the two-tailed Student’s *t*-test for unpaired samples. Differences in ascorbic acid and α-tocopherol concentrations in SP and serum, before and after administration of vitamin C or E, were evaluated by the two-tailed Student’s *t*-test for paired samples. A value of *p* < 0.05 was considered statistically significant.

## 3. Results

According to the results of the spermiogram, all fertile healthy controls could be classified into the normozoospermic category, according to the WHO classification (Table 1).

Concentrations of water-soluble antioxidants measured in both SP and serum of 15 fertile subjects are reported in Table 2.

Whist GSH and uric acid in SP (17.64 ± 4.12 and 232.37 ± 44.13 μmol/L, respectively) were approximately analogue to those measured in serum (15.54 ± 2.66 and 270.46 ± 57.90 μmol/L, respectively), concentrations of ascorbic acid in SP (286.01 ± 75.29 μmol/L) were significantly higher than those found in serum (57.52 ± 14.8 μmol/L, *p* < 0.001). Striking differences occurred when considering fat-soluble antioxidants in the two body fluids (Table 3).

Any of these compounds had significantly lower concentrations in SP than those detected in serum. The most abundant fat-soluble antioxidant α-tocopherol was 3.06 ± 0.85 μmol/L in SP and 28.51 ± 7.08 μmol/L in serum (*p* < 0.001). Similarly, much lower concentrations of all-*trans*-retinol (0.068 ± 0.028 μmol/L in SP and 5.69 ± 1.89 μmol/L in serum, *p* < 0.001), total carotenoids (0.108 ± 0.035 μmol/L in SP and 1.30 ± 0.28 μmol/L in serum, *p* < 0.001) and coenzyme Q_10_ (0.013 ± 0.006 μmol/L in SP and 0.152 ± 0.042 μmol/L in serum, *p* < 0.001) were recorded in SP samples. Biomarkers related to oxidative/nitrosative stress, reported in Table 4, were lower in SP than in serum (*p* < 0.001), even though the concentrations of MDA, nitrites and nitrates in the two fluids were of the same order of magnitude. Concentration of 8-OHdG in any SP or serum sample analyzed was below the limit of detection (0.001 μmol/L) of the HPLC method used.

**Figure 1:**
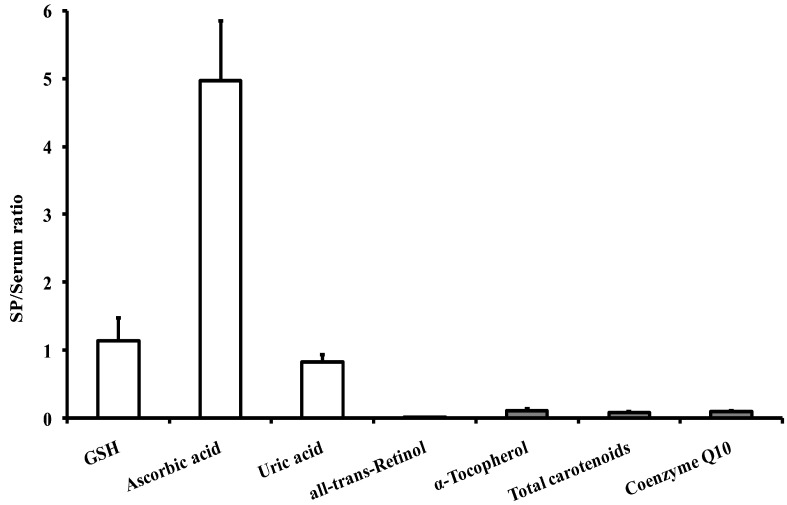
SP/serum ratios of water- and fat-soluble antioxidants calculated in 15 healthy fertile controls. Total carotenoids are the sum of the values of: astaxanthin, lutein, zeaxanthin, *trans*-β-apo-8′-carotenal, γ-tocopherol, β-cryptoxanthin, α-tocopherol, lycopene, α-carotene and β-carotene.

To better appreciate the different antioxidant profiles of the fluids under evaluation, the SP to serum ratios were calculated and illustrated in Figure 1.

It clearly evident that the only antioxidant having a higher concentration in SP than in serum is ascorbic acid, with an SP/serum ratio of 4.97 ± 0.88. In addition, it is also possible to note that the two other water-soluble antioxidants—GSH and uric acid—are equally distributed in the two fluids (SP/serum ratio = 1.14 ± 0.34 and 0.82 ± 0.12 for GSH and uric acid, respectively). As far as fat-soluble antioxidants are concerned, their SP/serum ratio ranged from 0.08 (total carotenoids) to 0.11 (α-tocopherol), i.e., the serum had about 10 times higher levels of fat-soluble antioxidants and vitamins. Among the various carotenoids detectable using the present HPLC method [43], the greatest discrepancies between the fluids were observed considering lycopene (SP/serum ratio = 0.014 ± 0.001), β-carotene (SP/serum ratio = 0.029 ± 0.006) and lutein (SP/serum ratio = 0.11 ± 0.02). It is also worth noting that the SP/serum ratio of α-tocopherol (0.11 ± 0.03) indicates that SP has a nine times lower concentration of the most important fat-soluble membrane antioxidant.

The SP/serum ratio of MDA, which is an indicator of ROS-mediated lipid peroxidation, was 0.33 ± 0.05 (*p* < 0.001) and those of –NO_2_^−^ and –NO_3_^−^, representative of nitric oxide production, were 0.66 ± 0.15 and 0.53 ± 0.09 (*p* < 0.001), respectively, i.e., the serum has two to three times higher concentration of oxidative/nitrosative biomarkers than SP.

To determine whether the daily administration of low doses of vitamin C (100 mg/day) or vitamin E (20 IU/day = 30 mg/day) for a sub-chronic period of 4 weeks might influence the corresponding concentrations of ascorbic acid and α-tocopherol in SP, we selected two subgroups of five subjects, each from the healthy fertile control group who had the lowest SP concentrations of either ascorbic acid or α-tocopherol.

**Figure 2:**
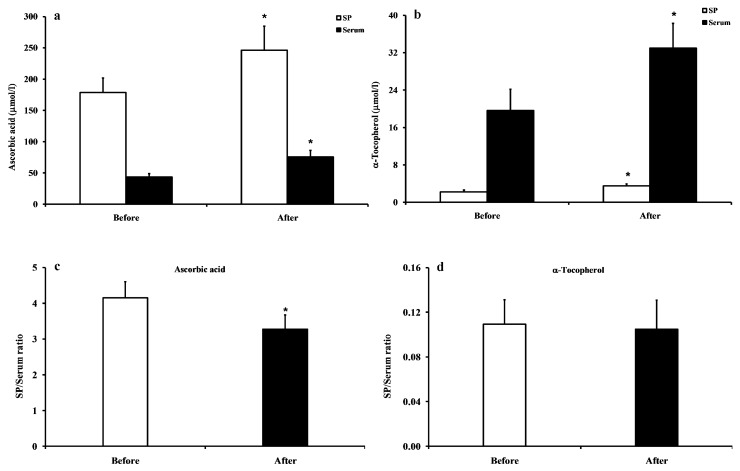
Concentrations of ascorbic acid (**a**) and α-tocopherol (**b**) in SP and serum, and SP/serum ratios of ascorbic acid (**c**) and α-tocopherol (**d**) before and after the daily administration of vitamin C or vitamin E (100 or 30 mg/day for 4 weeks) to two subgroups of fertile healthy controls who had the lowest SP values of ascorbic acid or α-tocopherol, respectively. * significantly different from the value determined before treatment, *p* < 0.01.

Figure 2a shows that the group of subjects with low SP ascorbic acid had concentrations, before and after treatment, of 178.65 ± 23.02 and 246.21 ± 38.24 μmol/L SP, respectively (*p* < 0.01). At the same time, the serum ascorbic acid in these subjects was 43.02 ± 5.66 and 75.14 ± 10.85 μmol/L (*p* < 0.01). Figure 2b indicates that the group of subjects with low SP α-tocopherol had concentrations, before and after treatment, of 2.14 ± 0.45 and 3.46 ± 0.38 μmol/L SP (*p* < 0.01), respectively. At the same time, α-tocopherol concentrations in the serum of these subjects were 19.58 ± 4.62 and 33.01 ± 5.27 μmol/L (*p* < 0.01).

Figure 2 gives evidence that the SP/serum ratio of ascorbic acid (**c**) was slightly but significantly decreased from 4.15 ± 0.45 before treatment to 3.28 ± 0.39 after treatment (*p* < 0.01), whilst the SP/serum ratio of α-tocopherol at the same time point (**d**) was unchanged (0.11 ± 0.022 before and 0.10 ± 0.026 after treatment).

## 4. Discussion

Results of the present study allowed evidence for the peculiar composition in water- and fat-soluble antioxidants of the SP of control fertile subjects compared with the antioxidant profile detectable in serum. According to the data of Table 2 and Table 3, human SP, being characterized by low concentrations of hydrophobic antioxidants, mainly relies on hydrophilic compounds to protect spermatozoa from ROS-mediated oxidative stress.

In our cohort of fertile healthy controls, we found that ascorbic acid concentration in SP was almost five times higher than that measured in their serum. This strongly suggests a mechanism of transport capable of accumulating ascorbic acid against a concentration gradient from serum (where it is in lower concentration) to SP (where a higher concentration is present). Since ascorbic acid cannot be synthesized by human beings, any tissue, cell and peripheral district of the body is dependent on the ingestion of vitamin C-containing foods in order to satisfy its own ascorbic acid requirement [44]. Efficient absorption of this molecule, mainly occurring through the activity of the SVCT1 Na^+^-ascorbic acid transporter [45,46], allows obtaining conspicuous ascorbic acid serum concentrations and its distribution by the blood stream to different tissue, cell and peripheral districts. The cellular uptake of ascorbic acid is primarily achieved via the action of the SVCT2 Na^+^-ascorbic acid transporter. SVCT2 is ubiquitously distributed in the different organs/tissues/cells and effects the electrogenic ascorbic acid transport exploiting the favorable extracellular/intracellular sodium gradient [47,48].

If this mechanism is suitable to perform ascorbic acid transport from the bloodstream to the extracellular fluid and then within cells, it appears rather implausible in the case of SP. To permit SP reaching ascorbic acid concentrations about five times higher than those in circulating blood (serum), cells of the seminal vesicles, prostate and bulbourethral glands (i.e., the main contributors, in a different percentage to the generation of SP) operate against a concentration gradient of both Na^+^ and ascorbic acid, since sodium concentration within SP is almost two times higher than that of plasma [48,49,50]. Therefore, the electrogenic process driven by the extracellular/intracellular Na^+^ concentration and coupled to the ascorbic acid cellular inlet [46,47], in cells other than those of seminal vesicles, prostate and bulbourethral glands, cannot be evoked in the accumulation of the molecule within SP. It is a conceivable hypothesis that either all or one of the aforementioned cells possess the ability to transfer ascorbic acid from serum to SP using active, energy-dependent mechanisms. Energy consumption would allow seminal vesicles, prostate and bulbourethral glands to release it in SP against a concentration gradient of both Na^+^ and ascorbic acid. To our knowledge, the two ascorbic acid carriers SVCT1 and SVCT2 (encoded by the *SLC23A1* and *SLC23A2* genes) do not effect this type of transport [45,46,47,48] which is, vice versa, operative in bacteria [51].

Differing from ascorbic acid, SP values of GSH and uric acid almost equaled those found in serum, indicating a free distribution of these molecules regulated on their relative concentrations in the two fluids. It is worth recalling that albeit both compounds are synthesized intracellularly, only uric acid is released and found in much higher amounts outside rather than inside cells [52].

Considering fat-soluble antioxidants, results demonstrate that their concentrations are 6 (all-*trans*-retinoic acid) to 70 (lycopene) fold lower in SP than in serum (in general, the more hydrophobic the molecule is, the less concentrated it is in SP). It is highly presumable that the low concentrations of α-tocopherol in SP (about nine times lower than in serum) render membranes of human spermatozoa particularly susceptible to ROS mediated lipid peroxidation, in the case of increased oxidative stress. However, since one of the roles of ascorbic acid is to regenerate α-tocopherol, it also reasonable to hypothesize that the high ascorbic acid concentration in SP might accelerate α-tocopherol recycling, thus mitigating the potentially negative effects of low levels of α-tocopherol in SP. When looking at the different levels of fat-soluble antioxidants in SP and serum, it is possible to affirm that none of these compounds is preferentially transported from serum to SP, strongly suggesting the lack of efficient carrier systems capable of accumulating higher concentrations in SP. The cumulative concentrations of fat-soluble antioxidants in the two fluids, calculated from the data reported in Table 3, return values of 3.32 and 37.34 μmol/L in SP and serum, respectively, demonstrating that only 1/11 of the circulating levels of these antioxidants is somehow transferred from serum to SP.

In this study, we also evaluated the influence of the serum concentrations on the SP levels of the main water- and fat-soluble antioxidants, i.e., ascorbic acid and α-tocopherol. The sub-chronic administration of low doses of either vitamin C (100 mg/day) or vitamin E (30 mg/day), to those fertile healthy controls who had the lower SP concentrations of ascorbic acid or α-tocopherol, respectively, produced significant increases in the levels of the two compounds in both SP and serum. However, when considering the SP/serum ratios, the striking diversities occurring between the two antioxidants allow us to confirm the hypothesis that there are different mechanisms regulating their passages from the blood stream to SP. Considering ascorbic acid—notwithstanding its concentrations raised from 178.65 ± 23.02 to 246.21 ± 38.24 μmol/L in SP and from 43.02 ± 5.66 to 75.14 ± 10.85 μmol/L in serum—the SP/serum ratio decreased from 4.15 ± 0.45 before treatment to 3.28 ± 0.39 after treatment. This finding strongly suggests that carrier mechanisms, rather than passive diffusion controlled by the concentrations in the two fluids, are involved in the accumulation process in which cells of the seminal vesicles, prostate and bulbourethral glands pump ascorbic acid within SP against a concentration gradient. On the other hand, α-tocopherol increased proportionally in both SP and serum, so that the SP/serum ratio before (0.11 ± 0.022) and after treatment (0.10 ± 0.026) was unchanged, thus indicating that the mechanism of transfer from serum to SP of this fat-soluble antioxidant is mainly dependent on its circulating concentrations. In other words, while in the case of α-tocopherol (and fat-soluble antioxidants in general) there is a strict relationship between its circulating and SP concentrations, this does not appear to be valid in the case of ascorbic acid. It is worth recalling that the peculiar transport of ascorbic acid in SP through a still-unknown mechanism is specific of this compound since the passage from serum to SP of the other highly concentrated water-soluble antioxidant (uric acid) appears to be performed by diffusion according to the concentration gradients of uric acid in the two fluids. It is worth underlining that in any of these mechanisms leading to the final antioxidant composition of SP, the role of seminal vesicles, prostate and bulbourethral glands is certainly fundamental and rather unusual since they are capable of allowing the passage of molecules, taking them up from serum and discharging them in SP using a plethora of different mechanisms, likely including active transport to ensure ascorbic acid accumulation in SP against a concentration gradient.

In the clinical setting, the administration of antioxidants to treat males affected by idiopathic infertility, representing about 35% of all infertile males [53], is a rather common practice. Various studies have been published reporting the beneficial effects of the oral administration of antioxidants to improve male fertility [54,55,56]. Surprisingly, in none of these studies have the water- and fat-soluble antioxidant profiles of SP and/or serum been measured. Thus, it is not clear whether those infertile males had deficits in one or more specific antioxidant. Additionally, there are no available data in the literature showing that the administration of a given antioxidant provokes the increase of its concentration in SP after treatment. In some instances, the determination of the so-called total antioxidant capacity (TAC) has been performed [57,58]. This gross evaluation of the antioxidant defenses present in a given biological sample does not permit an accurate qualitative and quantitative analysis of the different water- and fat-soluble antioxidants. Using TAC, or other similar gross evaluation, it is highly possible that, in the presence of decreased values of this parameter, the administration of the wrong antioxidant is performed.

According to our results, for males with idiopathic infertility it should be mandatory to perform analyses suitable to determine the full profiles of water- and fat-soluble antioxidants in SP and serum before any treatment with antioxidants, in order to establish whether specific deficits are present. It should also be recommended to repeat the analyses on SP after treatment to verify the effectiveness of the antioxidant administration. In the case of negative results after an antioxidant based therapy, it is plausible that the mechanisms underlying the transfer of that specific antioxidant are dysfunctional, rendering its administration useless. Hence, our analytical approach may have an important application in driving personalized therapies based on the SP and serum profiles of water-and fat-soluble antioxidants.

## 5. Conclusions

Results of this study showed the peculiar composition in low molecular weight antioxidants of human SP. The main defensive barrier is represented by water-soluble antioxidants, among which ascorbic acid is in concentration five times higher than in serum. This suggests the existence of still-unknown mechanisms allowing the accumulation of ascorbic acid within SP against a concentration gradient. The low amount of fat-soluble antioxidants in SP suggest that their administration to treat male infertility characterized by excess ROS production should be performed for a prolonged period of time and carefully monitored with an appropriate analysis capable of measuring quantitatively and qualitatively the whole antioxidant pattern of SP.

## Figures and Tables

**Table 1:** Morpho-functional parameters of semen in the spermiogram of fertile controls.

Age (Years)	Sperm Concentration (Million/mL)	Total Sperm Motility (%)	Sperm Morphology (% of Normal Spermatozoa)
39.12 ± 14.38 ^a^ (25–41)	128.54 ± 56.39 (108–195)	88.05 ± 10.44 (68–93)	76.62 ± 10.65 (69–84)

^a^ Values are the mean ± SD of 15 fertile healthy controls. Ranges of values of all parameters are given in parentheses.

**Table 2:** Concentrations of water-soluble antioxidants measured by HPLC in seminal plasma (SP) and serum of 15 healthy fertile volunteers.

Compounds	SP	Serum
**Glutathione (GSH)**	17.64 ± 4.12 ^a^ (13–22)	15.54 ± 2.66 (12–19)
**Ascorbic acid**	286.01 ± 75.29 ^b^ (205–350)	57.52 ± 14.81 (38–71)
**Uric acid**	232.37 ± 44.13 (193–290)	270.46 ± 57.90 (200–320)

^a^ Values are expressed as μmol/L and are reported as the mean ± SD. Concentration ranges of all parameters are given in parentheses. ^b^ Significantly different from values recorded in serum, *p* < 0.001.

**Table 3:** Concentrations of fat-soluble antioxidants measured by HPLC in SP and serum of 15 healthy fertile volunteers.

Compounds	SP	Serum
**all-*trans*-retinoic acid**	0.001 ± 0.001 ^a,b^ (0–0.002)	0.006 ± 0.003 (0.004–0.012)
**all-*trans*-retinol**	0.068 ± 0.028 ^b^ (0.030–0.080)	5.69 ± 1.89 (4.00–8.50)
**α-tocopherol**	3.06 ± 0.85 ^b^ (2.20–4.10)	28.51 ± 7.08 (15.50–34.00)
**γ-tocopherol**	0.066 ± 0.024 ^b^ (0.041–0.086)	1.68 ± 0.76 (0.65–2.20)
**Astaxhantin**	N.D.	0.004 ± 0.002 (0.003–0.008)
**Lutein**	0.067 ± 0.032 ^b^ (0.036–0.089)	0.593 ± 0.211 (0.330–0.770)
**Zeaxhantin**	0.001 ± 0.001 ^b^ (0–0.002)	0.009 ± 0.003 (0.003–0.013)
***trans*-β-apo-8′-carotenal**	0.022 ± 0.005 ^b^ (0.017–0.028)	0.152 ± 0.033 (0.080–0.185)
**β-cryptoxanthin**	0.003 ± 0.001 ^b^ (0.001–0.004)	0.019 ± 0.005 (0.012–0.029)
**Lycopene**	0.003 ± 0.001 ^b^ (0.001–0.004)	0.211 ± 0.096 (0.135–0.350)
**α-carotene**	0.004 ± 0.002 ^b^ (0.002–0.007)	0.035 ± 0.006 (0.028–0.048)
**β-carotene**	0.008 ± 0.005 ^b^ (0.003–0.140)	0.274 ± 0.099 (0.200–0.435)
**Total carotenoids**	0.108 ± 0.035 ^b^ (0.050–0.190)	1.30 ± 0.28 (0.98–2.00)
**Coenzyme Q_10_**	0.013 ± 0.006 ^b^ (0.008–0.020)	0.152 ± 0.042 (0.100–0.320)

^a^ Values are expressed as μmol/L and are reported as the mean ± SD. Concentration ranges of all parameters are given in parentheses. N.D. = Not Detectable. ^b^ Significantly different from values recorded in serum, *p* < 0.001.

**Table 4:** Concentrations of biomarkers of oxidative/nitrosative stress measured by HPLC in SP and serum of 15 healthy fertile volunteers.

Compounds	SP	Serum
**Malondialdehyde (MDA)**	0.005 ± 0.005 ^a,b^ (0.001–0.012)	0.015 ± 0.008 (0.005–0.022)
**Nitrites (–NO_2_^−^)**	2.76 ± 0.85 ^b^ (1.50–5.25)	4.19 ± 1.61 (2.20–7.80)
**Nitrates (–NO_3_^−^)**	22.63 ± 13.55 ^b^ (12.00–46.00)	42.34 ± 12.88 (24.00–76.00)
**8-hydroxy-2′-deoxyguanosine (8-OHdG)**	N.D.	N.D.

^a^ Values are expressed as μmol/L and are reported as the mean ± SD. Concentration ranges of all parameters are given in parentheses. N.D. = Not Detectable. ^b^ Significantly different from values recorded in serum, *p* < 0.001.
